# A clinician perspective on the treatment of chronic myeloid leukemia in the chronic phase

**DOI:** 10.1186/s13045-022-01309-0

**Published:** 2022-07-11

**Authors:** Valentin García-Gutiérrez, Massimo Breccia, Elias Jabbour, Michael Mauro, Jorge E. Cortes

**Affiliations:** 1 Servicio Hematología y Hemoterapia, Hospital Universitario Ramón y Cajal, Instituto Ramón y Cajal de Investigación Sanitaria (IRYCIS), Madrid, Spain; 2 Department of Translational and Precision Medicine, Sapienza University, Rome, Italy; 3 The University of Texas MD Anderson Cancer Center, Houston, TX USA; 4 Memorial Sloan Kettering Cancer Center, New York, NY USA; 5 Georgia Cancer Center, Augusta University, Augusta, GA USA

**Keywords:** Chronic myeloid leukemia, Tyrosine kinase inhibitors, First-line treatment, Treatment switching, Treatment-free remission

## Abstract

Tyrosine kinase inhibitors (TKIs) have vastly improved long-term outcomes for patients with chronic myeloid leukemia (CML). After imatinib (a first-generation TKI), second- and third-generation TKIs were developed. With five TKIs (imatinib, dasatinib, bosutinib, nilotinib, and ponatinib) targeting *BCR*::*ABL* approved in most countries, and with the recent approval of asciminib in the USA, treatment decisions are complex and require assessment of patient-specific factors. Optimal treatment strategies for CML continue to evolve, with an increased focus on achieving deep molecular responses. Using clinically relevant case studies developed by the authors of this review, we discuss three major scenarios from the perspective of international experts. Firstly, this review explores patient-specific characteristics that affect decision-making between first- and second-generation TKIs upon initial diagnosis of CML, including patient comorbidities. Secondly, a thorough assessment of therapeutic options in the event of first-line treatment failure (as defined by National Comprehensive Cancer Network and European LeukemiaNet guidelines) is discussed along with real-world considerations for monitoring optimal responses to TKI therapy. Thirdly, this review illustrates the considerations and importance of achieving treatment-free remission as a treatment goal. Due to the timing of the writing, this review addresses global challenges commonly faced by hematologists treating patients with CML during the COVID-19 pandemic. Lastly, as new treatment approaches continue to be explored in CML, this review also discusses the advent of newer therapies such as asciminib. This article may be a useful reference for physicians treating patients with CML with second-generation TKIs and, as it is focused on the physicians’ international and personal experiences, may give insight into alternative approaches not previously considered.

## Introduction

The global incidence of chronic myeloid leukemia (CML) in 2017 was 34,179, with a total of 24,054 CML-related deaths [1]. The advent of tyrosine kinase inhibitors (TKIs) has significantly changed the treatment landscape, improving outcomes for patients with CML; treatment with the first-generation TKI imatinib has improved the 8-year overall survival rate from 20 to 87% [2]. Six TKIs are approved and are commonly used for the treatment of CML: imatinib; the second-generation (2G) TKIs dasatinib, nilotinib, and bosutinib; the third-generation TKI ponatinib; and the novel, first-in-class TKI specifically targeting the ABL myristoyl pocket (STAMP inhibitor), asciminib [3–7]. With the currently available and emerging TKIs, patients with CML can have an average life expectancy near that of the general population [8, 9], and this has significantly increased the overall prevalence despite the relatively low incidence rate [10]. Although coming from the same drug class, each TKI differs in terms of efficacy, adverse events (AEs), and effectiveness against *BCR*::*ABL* mutations. Therefore, when deciding the ideal TKI therapy, many factors need to be considered by clinicians and patients.

In this review, we explore the circumstances under which clinicians would consider a 2G TKI. Using case studies developed by the authors of this review, and factoring in clinician experience, patient characteristics, and real-world considerations, we discuss treatment decisions on 2G TKIs. This review focuses on five topics in the treatment of CML: first-line (1L) treatment, early switching and considerations for monitoring optimal responses, clinical considerations for treatment-free remission (TFR), treatment of CML during the COVID-19 pandemic, and the impact of emerging TKIs.

## First-line treatment and TKI choice

First-line treatment decisions are complex and include disease- and patient-specific factors in addition to other factors such as availability, dosing schedule, cost, and the presence of any comorbidities. Although imatinib remains the most widely used TKI for newly diagnosed (ND) CML in chronic phase (CML-CP), clinical studies have shown how 1L use of 2G TKIs can improve response outcomes and decrease rate of disease progression. However, their use must be balanced against potential risks and costs.

### Case study 1

A 65-year-old male patient was diagnosed with high-risk CML (European Treatment and Outcome Study long-term survival [ELTS] score of 2.53 and Sokal score of 1.9) after his primary care clinician identified an elevated white blood cell count of 58 × 10^9^/L during a routine follow-up visit. At diagnosis, the patient had a spleen size of 5 cm below the costal margin and 8% of blasts in peripheral blood. A chromosome banding analysis documented only the Philadelphia chromosome. The patient is sedentary and overweight and has a long-standing history of diabetes mellitus not well controlled with metformin. He had myocardial infarction 5 years ago, but with no additional episodes and has since stopped smoking. The patient prefers a low-cost drug.

### Case study 1—clinicians’ considerations in treatment approach

Treatment strategies for CML increasingly focus on achieving a fast, sustained, deep molecular response (DMR; molecular response with a 4.5-log reduction in *BCR*::*ABL1* [MR^4.5^] on the International Scale) with 1L treatment. The National Comprehensive Cancer Network (NCCN) guidelines and European LeukemiaNet (ELN) recommendations consider achievement of an early molecular response (EMR; *BCR*::*ABL1* ≤ 10% within 3 months) a treatment milestone for an optimal response; therefore, this is an important consideration when choosing a 1L TKI.

#### Clinical trial-based considerations: risk score and efficacy

The NCCN guidelines and ELN recommendations suggest 1L 2G TKIs for patients with low- or possibly intermediate-risk scores based on their better response outcomes over imatinib [11]. Moreover, the ELN recommendations suggest using the new ELTS scoring system to assess baseline CML [11], instead of Hasford/Sokal risk scores, as it predicts disease-specific mortality and molecular responses [12]. Although patients enrolled on key trials were used to develop the ELTS scoring system, the ELTS score has not yet been used prospectively in pivotal trials of 1L TKIs. The NCCN guidelines recommend using the Sokal score [13], which is also widely used outside of the USA, but the ELTS score is useful for assessing long-term survival outcomes, especially for younger patients. As clinicians, we often use both scoring systems.

Key clinical trials, DASISION (dasatinib vs. imatinib), ENESTnd (nilotinib vs. imatinib), and BFORE (bosutinib vs. imatinib), in which most patients had intermediate- or high-risk CML, showed superior rates of complete cytogenetic responses (CCyR) and major molecular responses (MMR) with 1L dasatinib, nilotinib, and bosutinib over imatinib (Table 1) [14–16]. MMR at 12 months was significantly higher with all three 2G TKIs compared with imatinib and was sustained over long-term follow-up [17–19]. However, none of the 2G TKIs (except for nilotinib at 400 mg) resulted in a significant improvement in overall survival or progression-free survival compared with imatinib, meaning earlier responses and enhanced response rates with 2G TKIs did not necessarily lead to improvements in overall survival and progression-free survival. Furthermore, approximately 40–60% of patients treated with 2G TKIs were unlikely to achieve MR^4.5^, with a plateau observed at approximately 5 years [17, 19, 20].

**Table 1:** Efficacy of 2G TKIs in newly diagnosed CML-CP

Trial	Inclusion criteria	Key efficacy data	Safety	Treatment discontinuations and reasons
*Dasatinib. DASISION* (NCT00481247) [17]: phase 3 multicenter trial comparing dasatinib with imatinib in the first-line treatment of ND Ph + CML-CP	Aged ≥ 18 years Newly diagnosed Ph + CML-CP ECOG PS 0–2 No prior TKI treatment* No baseline pleural effusion Select CV conditions not excluded: myocardial infarction > 6 months, congestive heart failure > 3 months, or uncontrolled angina > 3 months prior to enrollment	Confirmed CCyR by 12 months (primary endpoint): Dasatinib 77%; imatinib 66% (*P* = 0.007) MMR at 12 months: Dasatinib 46%; imatinib 28% (*P* = 0.0001) Cumulative 5-year MMR rate: Dasatinib 76%; imatinib 64% (*P* = 0.002) Cumulative 5-year MR^4.5^ rate: Dasatinib 42%; imatinib 33% (*P* = 0.0251)	Drug-related pleural effusion: Dasatinib 28%; imatinib 1%	Discontinuations: Dasatinib (*n* = 100, 39%) Imatinib (*n* = 96, 37%) Dasatinib: Intolerance (*n* = 42, 16%) Progression or treatment failure (*n* = 28, 11%) Imatinib: Progression or treatment failure (*n* = 36, 14%) Intolerance (*n* = 17, 7%)
*Nilotinib. ENESTnd *(NCT00471497) [18]: phase 3, randomized, open-label, multicenter trial of nilotinib (300 mg or 400 mg BID) versus imatinib in patients with ND Ph + CML-CP	Aged ≥ 18 years Ph + CML-CP diagnosed within 6 months of diagnosis ECOG PS 0–2 No cardiovascular conditions No T315I mutations	MMR at 12 months (primary endpoint): Nilotinib 300 mg 44%; nilotinib 400 mg 43%; imatinib 22% (*P* < 0.001 nilotinib vs. imatinib) 5-year MR^4.5^: Nilotinib 300 mg 54% (*n* = 151/279); nilotinib 400 mg, 52% (*n* = 147/277); imatinib 31% (*n* = 89/280) (*P* < 0.0001 nilotinib vs. imatinib) Estimated progression-free survival: Nilotinib 300 mg 96%; imatinib 91% (*P* = 0.0204) Estimated overall survival: Nilotinib 300 mg, 96%; imatinib, 92% (*P* = 0.0266)	Grade 3/4 cardiovascular events: Nilotinib 300 mg 5% (*n* = 13/279); nilotinib 400 mg 9% (*n* = 24/277); imatinib 2% (*n* = 5/280) Grade 3/4 elevated glucose levels: Nilotinib 300 mg 7% (*n* = 20/279); nilotinib 400 mg 7% (*n* = 19/277); imatinib < 1% (*n* = 1/280)	Imatinib discontinuations (*n* = 139): Suboptimal response/treatment failure (*n* = 59) AEs/abnormal laboratory values (*n* = 38) Nilotinib 300 mg discontinuations (*n* = 110): AEs/abnormal laboratory values (*n* = 34) Suboptimal response/treatment failure (*n* = 34) Nilotinib 400 mg discontinuations (*n* = 105): AEs/abnormal laboratory values (*n* = 56) Withdrawal of consent (*n* = 16) Suboptimal response/treatment failure (*n* = 13)
*Bosutinib. BFORE *(NCT02130557) [15, 25, 102, 103]: phase 3, randomized, open-label, multicenter trial of bosutinib versus imatinib in patients with ND Ph + or Ph-/*BCR*::*ABL1* + CML-CP	Aged ≥ 18 years Newly diagnosed Ph + or Ph-/*BCR*::*ABL1* + CML-CP (≤ 6 months from initial diagnosis) ECOG PS 0–1 No prior treatment, including TKIs	MMR at 12 months (primary endpoint): Bosutinib 47%; imatinib 37% (*P* = 0.0200) CCyR at 12 months: Bosutinib 77%; imatinib 66% (*P* = 0.0075)	Grade ≥ 3 vascular events by 18 months: Bosutinib 2%; imatinib 0% Most common grade ≥ 3 non-hematologic TEAEs: Increased alanine transaminase: bosutinib 21%; imatinib 2% Increased aspartate aminotransferase: bosutinib 10%; imatinib 2%	Bosutinib discontinuation at 12 months (*n* = 59/268, 22%): AEs (*n* = 37, 14%) Patient request (*n* = 6, 2%) Imatinib discontinuations at 12 months (*n* = 71/265, 27%): AEs (*n* = 24, 9%) Suboptimal response/treatment failure (*n* = 16, 6%)
*Nilotinib and dasatinib. JALSG CML212* (#UMIN000007909) [104]: phase 3, randomized, open-label, multicenter trial of achievement of MR^4.5^ after treatment with nilotinib versus dasatinib	Newly diagnosed CML-CP confirmed by cytogenetic study and/or detection of *BCR*::*ABL1* by RT-PCR	Cumulative MR^4.5^ rates at 18 months (primary endpoint): Nilotinib 33%; dasatinib 31%	Grade 3/4 AEs with ≥ 10% frequency nilotinib: Lipase elevation (12%) Grade 3/4 AEs with ≥ 10% frequency dasatinib: Thrombocytopenia (17%) Neutropenia (13%)	Discontinued treatment by 18 months: 24% of nilotinib- and 20% of dasatinib-treated patients

*2G* second generation; *AE* adverse event; *CCyR* complete cytogenetic response; *CML-CP* chronic myeloid leukemia chronic phase; *CV* cardiovascular; *ECOG PS* Eastern Cooperative Oncology Group performance status; *MMR* major molecular response; *MR*^4.5^ 4.5-log reduction in *BCR*::*ABL1*; *ND* newly diagnosed; *Ph*+ Philadelphia positive; and *TEAE* treatment-emergent adverse event

*Prior TKI allowed for required disease management while awaiting study start; commercial supplies of Gleevec (Glivec) at any dose could be prescribed, but for no longer than 2 weeks in duration

Because the patient in this case study has high-risk CML, a 2G TKI would be best suited for 1L treatment. Regardless of risk score, younger patients [21] and those with rare transcripts [22] should also be considered as high-risk, and a 2G TKI may be the preferred option. Patients with low-risk CML may also benefit from 1L 2G TKI treatment; in the 5-year report of the BFORE trial, the MR^4.5^ rate for patients with a low Sokal risk score was 53.7% with bosutinib versus 42.5% with imatinib [19]. Thus, not only should choice of TKI be based on risk score and efficacy, it should also be tailored to each patient and balanced against the goals of therapy.

#### Clinical trial-based considerations: comorbidities

The use of 1L 2G TKIs must be balanced against their potential risk; therefore, clinicians should also consider the known AEs associated with 2G TKIs and baseline patient comorbidities. Patient comorbidities are often assessed using the Charlson Comorbidity Index (CCI), and survival of patients with CML decreases with increasing CCI score [18], with the risk of death driven mostly by comorbidities [23]. In DASISION, dasatinib was associated with improved outcomes over imatinib across all CCI subgroups, with a significant difference in MR^4.5^ rates with a higher comorbidity score suggesting sustained efficacy of dasatinib, even among patients with a substantial comorbidity burden [24].

Certain cardiovascular (CV) and arterio-occlusive events (AOEs) were more common in patients treated with dasatinib, nilotinib, and bosutinib than imatinib [15, 16, 25]. Dasatinib is associated with an increased risk of developing pulmonary hypertension and cardiac dysfunction compared with imatinib [5, 14]; patients with hypertension treated with dasatinib are at an increased risk of developing pleural effusions [26, 27]. Nilotinib and dasatinib may affect the QT interval [4, 5], and nilotinib especially poses a greater risk of both CV and cerebrovascular AOEs compared with imatinib [18]. A lower relative risk of AOEs was observed with bosutinib but was still higher than with imatinib [25]. Once a 2G TKI is considered the best option, close monitoring and aggressive management of comorbidities and other risk factors (e.g., smoking, diet, sedentarism) are important to minimize the risk of AOEs.

Commonly, patients with CML are overweight, which is often associated with CV disease and diabetes mellitus [28]. Although imatinib has shown reduced efficacy in overweight patients [29], responses in overweight patients treated with dasatinib are comparable to patients with a normal weight, with a surprisingly faster median time to MMR achieved in overweight patients [30]; however, overweight patients were at a higher risk of pleural effusion than patients with a normal weight (34% vs. 25%) [30]. Being overweight did not affect responses with bosutinib, but overweight patients treated with bosutinib had increased levels of alanine and aspartate aminotransferase compared with patients with normal weight [31].

Baseline monitoring for diabetes and renal/liver disease is also important when choosing 2G TKIs. Hyperglycemia and an increased risk of developing prediabetes has been associated with nilotinib treatment [32], and bosutinib and nilotinib can cause an elevation in alanine transferase and lipase levels [4, 6]. Additionally, bosutinib and imatinib have been associated with a reversible decrease in glomerular filtration rate [33], although this likely does not represent kidney damage in most instances. In patients presenting with multiple comorbidities, it is important to consider carefully the potential increased risks associated with a 2G TKI while also ensuring that the patient can achieve the best possible outcome.

#### Considerations based on real-world evidence: costs and adherence

Imatinib is now available as a generic drug in several countries; however, access to dasatinib, bosutinib, and nilotinib is limited in many low- and middle-income countries, partly due to regulatory authorities mandating imatinib as first choice [34] or lack of financial support for paying the cost of brand-named drugs. Moreover, the costs incurred through lost wages and travel for medical appointments have an impact even when the cost of the TKI is subsidized through treatment assistance programs, especially in low- and middle-income countries [35].

Adherence to treatment is crucial for improvements in response rates and survival. In patients treated with imatinib, an adherence rate of > 90% correlated with a 6-year probability of achieving a DMR (4-log reduction in *BCR*::*ABL1* [MR^4^]) of 76% versus 4% (*P* < 0.001) [36]. Real-world evidence from low- and middle-income countries demonstrated that a decreased adherence to imatinib treatment was associated with lower 10-year event-free survival [37]. Increased adherence has also been associated with reduced hospitalization costs and fewer hospital admissions [38–42].

Numerous factors affect adherence: AEs, disease and treatment duration, forgetting to take medication, inconvenience of medication frequency, cost, lack of engagement, and disease-related education [43–45]. Increased *BCR*::*ABL1* monitoring, lower co-payments, and fewer daily doses all correlate with increased adherence [42, 45, 46]. Overall, patient education on the impact of adherence and regular communication between the clinician and patient regarding AE management and financial issues are important for optimizing adherence. Monitoring patient-reported outcomes can help to identify early subtle changes that may affect patient’s adherence to therapy and/or overall wellbeing.

#### Treatment approach summary

The patient described in case study 1 has significant comorbidities that increase the risk of developing AOEs, which need to be carefully weighed against his treatment goals. Given the relatively high survival rate, imatinib may be an adequate choice if the cost of TKIs is not supported by government assistance programs. If the patient’s goals include deeper responses and achieving TFR, then a 2G TKI would be a better candidate.

Because this patient has a high CV risk, the likely best TKI within this context would be bosutinib; despite the low percentages, CV events were reported at a higher rate with nilotinib and dasatinib compared with imatinib at 5 years [17, 18]. If this patient were to have a history of certain lung diseases such as interstitial pneumonitis, dasatinib should be avoided. As this patient has a history of diabetes mellitus, dasatinib should be considered due to its lower risk of metabolic effects compared with bosutinib or nilotinib [4–6]. The higher probability of sustained MR^4.5^ with nilotinib versus imatinib should be balanced with the risk of CV events.

AOEs are seldom fatal, as seen in one study reporting only three deaths with nilotinib (*n* = 563) and none with imatinib (*n* = 283) [20]. Without appropriate management of comorbidities, the likelihood of having an AOE and death from comorbidities might be higher than from CML. Aggressive management of comorbidities and the necessary behavioral changes (e.g., diet, exercise) will optimize survival outcomes from all causes. This patient should commit to these behavioral changes if treated with bosutinib.

## Early switching of TKI

Throughout treatment, patients are monitored regularly to assess *BCR*::*ABL1* transcript levels in response to TKI therapy. Achievement of EMR with imatinib and 2G TKIs in the 1L setting is a predictor of DMR and improved survival in patients with CML-CP; therefore, it is an important treatment milestone. When EMR is not achieved with a 1L TKI, switching therapies can be considered. However, the timing of switching TKIs remains a controversial topic, with each clinician having their own approach. Regular monitoring of the initial response is associated with better outcomes as it ensures prompt switching in the case of intolerance/resistance. An earlier switch to 2G TKIs offers the hope of improved outcomes over a later switch in patients who fail to achieve an EMR on 1L treatment.

### Case study 2

A 56-year-old female patient with intermediate-risk CML (as assessed by ELTS score) was treated with imatinib (400 mg) and achieved a hematologic response after 3 weeks. During treatment, the patient experienced grade 2 skin rash and moderate fluid retention; the treatment was withheld for 10 days until toxicities resolved. Once toxicities resolved, she resumed treatment with a lower dose of imatinib (300 mg). A dose increase was attempted but moderate fluid retention reappeared; therefore, imatinib was maintained at 300 mg. Treatment evaluation at 3 months showed inadequate response with a *BCR::ABL1* level of 26%.

### Case study 2—clinicians’ considerations in treatment approach

#### Clinical trial-based considerations

The ELN recommendations and NCCN guidelines classify treatment failure as a confirmed lack of EMR [11, 13]. Confirmation is especially important when cytogenetic response is not monitored and/or *BCR*::*ABL1* levels are close to 10%.

Second-line (2L) treatment with dasatinib and nilotinib can result in high MMR rates in patients with an inadequate response to imatinib [47]. Patients who achieved deep responses early on in treatment were shown to have more favorable long-term outcomes over those who achieved similar responses later on in treatment, highlighting the importance of early versus late switching [47, 48].

DASCERN was the first prospective trial to demonstrate the potential benefit of early switching to dasatinib in patients who did not achieve EMR after 3 months of treatment with imatinib [49]. Patients who switched to dasatinib at 3 months had a significantly higher MMR rate at 12 months than patients who remained on imatinib (29% vs. 13%, *P* = 0.005); and cumulatively by month 24, more patients on dasatinib had achieved MMR (64% vs. 41%) once treatment crossover was accounted for [49]. Additionally, findings from the LASOR trial suggested that patients with a suboptimal cytogenetic response (per the less stringent ELN 2009 recommendations [50]) at 3 months were more likely to achieve improved cytogenetic and molecular responses with switching to nilotinib than with imatinib dose escalation (CCyR at 6 months: 50% vs. 36%, nominal *P* = 0.058), although the difference was not statistically significant when responses achieved after crossover were included [51].

Once treatment failure is identified, the probability of achieving DMRs after switching to a 2G TKI decreases, while the likelihood of disease progression increases. In patients from the DASISION and ENESTnd trials, who did not achieve EMR at 3 months and experienced disease progression, approximately half progressed between 3 and 6 months after treatment failure was identified [17, 52]. Findings from these clinical trials provide new insights into the potential benefit of switching to 2G TKIs in patients failing to achieve EMR with 1L imatinib. However, longer follow-up is warranted to determine if earlier responses after switching would result in improvements in survival outcomes, which may be more clinically meaningful.

#### Real-world evidence and real-world considerations for monitoring responses to TKIs

ELN recommendations state that *BCR*::*ABL1* mutational analysis must be performed in order to continue treatment with the most effective TKI [11]. However, the retrospective observational TARGET-UK study, which evaluated baseline monitoring practices in UK patients with ND CML-CP, found that ELN monitoring recommendations were not consistently implemented [53]. This left patients at a higher risk of relapse: 23% of patients with ELN-defined treatment failure did not switch treatment, and only 49% of patients who switched due to treatment failure had undergone mutation analysis [53].

In the ongoing observational SIMPLICITY study, treatment switching occurred in 16% of patients within 12 months of initiating treatment with 1L imatinib, dasatinib, or nilotinib [54]. More patients switched at months 3–12 (69%) than within 3 months (31%), with switching more common in patients treated with imatinib than dasatinib or nilotinib [54, 55]. The primary reasons for switching were intolerance and resistance, both more frequent with imatinib than with dasatinib (intolerance: 42% vs. 29%; resistance: 73% vs. 14%) [54, 55].

A retrospective analysis by the Italian Medicines Agency showed that within the first year of treatment with 2G TKIs, 7% of patients switched treatment (dasatinib: 8%; nilotinib: 7%); over a 6-year period, a total of 16% of dasatinib- and 11% of nilotinib-treated patients switched TKI. The primary reasons for switching were intolerance (59%) or resistance (57%), with most patients switching therapy within the first 12 months of treatment [56]. No specific baseline characteristics were associated with intolerance, but male patients appeared more likely to switch treatment due to resistance [56]. Treatment switching with a 1L 2G TKI was relatively uncommon and occurred at a much lower frequency in the Italian Medicines Agency study than the SIMPLICITY study [54–56]. For most patients with treatment failure, ponatinib was the preferred 2L option, but the median time to treatment change was 354 days. Overall, the frequency of switching was lower in real-world evidence studies compared with clinical trials [17–19], possibly due to trial protocols requiring patients to switch treatment once inadequate response was observed.

In patients treated with a 1L 2G TKI who lack an EMR, changing treatment should be taken with care. For patients requiring a treatment change due to intolerance, switching to a different 2G TKI and/or considering lower treatment doses might be the best option. Ponatinib appears to be the preferred next treatment choice for patients with treatment failure to dasatinib, nilotinib, or bosutinib; however, this recommendation is based on data from a setting where ponatinib was used in the third line and beyond. At the time of this review, there are limited prospective data on 2L therapy after resistance to a 2G TKI, with one recent observational study showing favorable efficacy with the use of 2L ponatinib [57].

#### Treatment approach summary

Successful management of CML may require careful selection of the initial TKI along with regular monitoring of responses and intolerance. Although monitoring is often underutilized, it is important for informing decisions on changes in therapy to minimize the risk of progression after lack of EMR with 1L therapy. Identifying early signs of intolerance or treatment failure, followed by early switching where necessary, may be important for ensuring the best outcome for patients.

For the patient outlined in case study 2, treatment failure is evident; therefore, treatment should be changed in a timely manner to minimize the risk of disease progression and increase the probability of optimal outcomes. Because the patient was treated with 1L imatinib, a switch to a 2G TKI would be appropriate. If there were no contraindications, dasatinib would be recommended based on the DASCERN study [49], with a change to nilotinib advised based on the LASOR study [51] if the patient was intolerant to dasatinib. Due to this patient’s history of fluid retention and the association of pleural effusion with dasatinib, switching to dasatinib is not recommended. However, nilotinib could be considered based on the low rates of edema reported with this agent [18]. Although ponatinib has demonstrated efficacy in patients with CML who are resistant/intolerant to 2G TKIs and those with the T315I mutation [58, 59], generally it is recommended for the treatment of CML in these patients [11, 13]. As the patient in this case study does not harbor the T315I mutation, we do not recommend ponatinib in this instance.

## Treatment-free remission

Indefinite use of TKIs is a common initial approach when treating patients with CML, regardless of response [13, 50, 60]. The achievement of sustained DMR on therapy is now considered a relevant clinical endpoint for patients who ultimately wish to stop treatment, thereby attempting TFR. Collective provisional guidance states that during TFR, patients who were treated with a minimum of 5 years of imatinib, or 3 years for 2G TKIs, and achieved sustained DMR for at least 2 years (measured by a reduction in *BCR*::*ABL1* level [International Scale] to ≤ 0.01% [MR^4^], ≤ 0.0032% [MR^4.5^], or ≤ 0.001% [5-log reduction in *BCR*::*ABL1*] [11, 13]) can stop TKI therapy. During TFR, regular monitoring (monthly for the first 6 months, then bimonthly thereafter [11, 13]) of *BCR*::*ABL1* levels is required, with the aim of maintaining very low or undetectable level of residual disease (threshold for relapse is MMR) [11, 13]. Further investigation is needed to identify strong predictors of successful TFR.

The depth of response required for TFR varied across different clinical trials examining TFR. In EURO-SKI, a minimum response of MR^4^ was required before a TFR attempt [61], but the probability of remaining treatment-free appeared higher with more stringent criteria (STIM, TWISTER, A-STIM) and was associated with a more stable plateau in the response curve [62–64]. In addition, an increased duration of DMR prior to TFR attempt was associated with a lower probability of relapse [65].

### Case study 3

A 33-year-old female patient with low-risk CML (as assessed by ELTS score) was treated with 1L imatinib with excellent tolerability. The patient achieved MMR after 12 months of treatment, followed by a sustained DMR for 4 years. She would like to discontinue imatinib as she is considering becoming pregnant.

### Case study 3—clinicians’ considerations in treatment approach

#### First treatment-free remission attempt (TFR1)—advantages and disadvantages of treatment-free remission

An overview of the results from the key trials on TFR can be found in Table 2. TFR after sustained DMR with 1L imatinib has been studied in the STIM and A-STIM trials [62, 64], in which approximately 40–50% of patients were able to sustain TFR for up to 7 or more years. Patients treated with 2G TKIs have also been able to achieve TFR. In DASFREE (Table 2), the largest clinical trial to date examining TFR in patients who discontinued dasatinib across all lines of therapy, 48% of patients who discontinued dasatinib maintained TFR after 1 year, and remission was durable at 2 years (one late relapse at month 39) [66]. Patients who lost MMR and restarted dasatinib quickly regained their response (median time to regain MMR and MR^4.5^ was 2 and 3 months, respectively) [66]. Additionally, dasatinib discontinuation was shown to be feasible in the D-STOP trial (63% of patients maintained MR^4^ after 1 year) and the phase 2 Japanese Dasatinib Discontinuation trial (estimated 3-year TFR rate of 44%) [67, 68].

**Table 2:** Summary of clinical trials examining treatment-free remission

Trial	Patient population	Key efficacy data	Key safety data
*Dasatinib DASFREE* (NCT01850004) [66]: phase 2 trial of dasatinib therapy discontinuation in patients with CML-CP and stable MR^4.5^	Aged ≥ 18 years Dasatinib treatment for ≥ 2 years as first-line or subsequent CML-CP therapy Dasatinib-induced DMR (MR^4.5^) for ≥ 1 year prior to enrollment ECOG PS 0–1	TFR at 12 months (primary endpoint), overall: 48% Discontinuation after 1L dasatinib: 54% Discontinuation after 2L + dasatinib: 43% MMR at 24 months (secondary endpoint): 45%	11% of patients (*n* = 9) experienced investigator-determined withdrawal events, including musculoskeletal pain and arterial hypertension
*Nilotinib ENESTop* (NCT01698905) [69]: discontinuation of nilotinib in patients with *BCR*::*ABL* + CML-CP who have achieved sustained MR^4.5^ with nilotinib after imatinib	Aged ≥ 18 years 2L nilotinib for ≥ 2 years following imatinib *BCR*::*ABL* + CML-CP Lack of MR^4.5^ on imatinib Achieved MR^4.5^ on nilotinib ECOG PS 0–2	TFR at 48 weeks (primary endpoint): 58%	Musculoskeletal pain within first 48 weeks of TFR reported in 42% of patients
*Nilotinib ENESTfreedom* (NCT01784068) [70]: discontinuation of nilotinib in patients with b3a2/b2a2 CML-CP who have achieved sustained DMR with 1L nilotinib	Aged ≥ 18 years 1L nilotinib for ≥ 2 years CML-CP with b3a2 and/or b2a2 MR^4.5^ at screening ECOG PS 0–2	TFR (MMR) at 48 weeks (primary endpoint): 52%	Musculoskeletal pain in first 48 weeks reported in 34% of patients
*Nilotinib ENESTgoal* (NCT01744665) [105]: discontinuation of nilotinib in patients with CML-CP who have achieved sustained MR^4.5^ after switching to nilotinib	1L imatinib for ≥ 1 year CML-CP with MMR but not MR^4.5^ Real-time qualitative polymerase chain reaction every 3 months	mRFS at 6 months: 7/17 patients sustained MR^4.5^	18% of patients experienced AEs during TFR
*Imatinib STIM* (NCT00478987) [62]: observational study of CMR persistence after discontinuing imatinib therapy	Aged ≥ 18 years CML-CP CMR under treatment with imatinib for ≥ 2 years No prior treatments: immunomodulatory (except interferon α), autologous hematopoietic stem cell transplantation, or for other malignancies	TFR: 6 months: 43% 60 months: 38%	No musculoskeletal pain was reported 1 patient progressed to lymphoid blast crisis after relapsing and resuming TKI treatment
*Imatinib A-STIM* (NCT02897245) [64]: observational study of MMR persistence after discontinuing imatinib therapy	CML-CP Treatment with imatinib CMR under treatment DMR (*BCR*::*ABL1* International Scale ≤ 0.01%)	TFR without loss of major molecular response (primary outcome): 1 year: 57% 3 year: 53% 5 year: 51% 7 year: 46%	No safety outcomes reported
*1L TKI EURO-SKI* (NCT01596114) [61]: phase 3, multicenter, open-label trial evaluating the persistence of MR in patients with CML after TKI discontinuation	Aged ≥ 18 years CML-CP with 1L TKI treatment or 2L if switched due to toxicity of 1L TKI ≥ 3 years of prior TKI therapy ≥ MR^4^ for ≥ 1 year	mRFS (primary endpoint): 6 months: 61% 24 months: 50% 49% patients lost MMR after TKI discontinuation	4 deaths unrelated to CML: 1 of each: myocardial infarction, lung cancer, renal cancer, and heart failure 6 deaths unrelated to CML-CP after loss of MMR and treatment re-initiation
*1L TKI DESTINY* (NCT01804985) [80]: UK, phase 2, open-label, multicenter trial of TKI de-escalation and stopping in patients with an excellent response to TKI treatment	Aged ≥ 18 years *BCR*::*ABL1* + CML-CP ≥ 3 years of prior TKI therapy ≥ 3 qualitative polymerase chain reaction transcripts of < 0.1% *BCR*::*ABL1* in the 12 months preceding enrollment	Relapse-free survival after 12 months de-escalation and 2 years of treatment discontinuation (primary endpoint): Patients with MR^4^ at trial entry: 72% Patients with MMR at trial entry: 36%	2 deaths due to unrelated causes

*1L* first-line; *2L* second-line; *AE* adverse event; *CCyR* complete cytogenetic response; *CML-CP* chronic myeloid leukemia in chronic phase; *CMR* complete molecular response; *ECOG PS* Eastern Cooperative Oncology Group performance status; *EMR* early molecular response; *MMR* major molecular response; *MR*^4^ 4-log reduction in *BCR*::*ABL1*; *MR*^4.5^ 4.5-log reduction in *BCR*::*ABL1*; *mRFS* molecular relapse-free survival; *TFR* treatment-free remission; and *TKI* tyrosine kinase inhibitor

Successful TFR has also been demonstrated with 1L and 2L nilotinib: 47% and 48% of patients discontinuing 1L and 2L nilotinib, respectively, remained in TFR at 144 weeks in the ENESTfreedom and ENESTop trials (Table 2) [69, 70]. Furthermore, in the STOP 2G TKI trial, which monitored TFR after dasatinib and nilotinib discontinuation, 63% of patients remained in TFR at 1 year [71]. Although the risk of relapse is highest during the first 6 months and decreases significantly after 2 years, late relapses can occur; approximately 15% of relapses occurred after 2 years [64], with relapses rare but possible in the blast phase [72]. Treatment cessation in TFR can reduce TKI-associated AEs, improving quality of life and decreasing treatment costs [61]. However, discontinuing TKI therapy can result in TKI withdrawal syndrome (mainly musculoskeletal pain), and patients require regular monitoring of *BCR*::*ABL1* levels [11, 13, 66, 69, 70]. Additionally, studies evaluating the psychological issues associated with TFR have shown that not all patients eligible to attempt TFR were comfortable to discontinue treatment due to fears of relapse or commitment to regular, frequent *BCR*::*ABL1* monitoring [73]. The improvements in quality of life have also been modest and inconsistent across various studies [74].

In our experience, a significant proportion of patients are interested in treatment discontinuation, and this should be discussed from diagnosis onward, with TFR only attempted after careful consideration, discussion, and assessment by clinicians.

#### First treatment-free remission—factors influencing the success of treatment-free remission

A longer duration of imatinib treatment prior to TFR was associated with a lower risk of relapse [61–63]. As treatment with 1L 2G TKIs demonstrates a faster, deeper response, it is possible that patients can attempt TFR after a shorter exposure to 2G TKIs than imatinib. In the ENESTfreedom study, patients attempting TFR after treatment with nilotinib for 3.5 years had similar TFR rates to patients treated with imatinib for more than 6 years [61, 62]. The optimal duration of DMR before attempting TFR is yet to be elucidated, although an increased time in DMR prior to entering TFR has been shown to increase the probability of maintaining MMR at 6 months [61, 65].

The effect of Sokal risk score on the success of TFR is under investigation; however, in the TWISTER and STIM-1 trials, a higher Sokal risk score was associated with a lower TFR success rate [62, 63]. Other factors possibly contributing to the success of TFR include older age [75, 76], minimal fluctuations in *BCR*::*ABL1* levels [64], and maintenance of MR^4.5^ in the first 3 or 4 months post-TFR [70, 77]. Advances in polymerase chain reaction methodology may allow earlier detection of relapse [78] and better identification of eligible patients for TFR [79]. Dose reduction prior to TFR attempt (based on the DESTINY study) [80] may decrease the risk of withdrawal syndrome. Furthermore, recent studies indicate that natural killer cells can be potential biomarkers for predicting the success of TFR [81, 82].

Not all patients are eligible for TFR, including those who have experienced disease progression to acute or blast phase, even if they have since reverted to CML-CP and regained DMR [12]; those who cannot be monitored frequently; and those with atypical transcripts that cannot be quantitated and therefore properly monitored. Achieving TFR in patients who are pregnant or who have a desire for pregnancy remains a controversial topic. Some clinicians prefer to attempt TFR before pregnancy in case of relapse, while others are inclined toward transient treatment interruptions or full TFR attempts during pregnancy [11, 13]. Based on available evidence, contraception is suggested for patients of child-bearing potential, and pregnancy should be planned only after stable response is reached [11]. Therefore, TFR may be an important treatment goal for patients of child-bearing potential.

The consensus on TFR in clinical practice is still evolving, but results from ongoing TFR trials will provide more confirmatory data on long-term outcomes. To date, TFR is successful in just 20–30% of patients treated with TKIs [61, 62, 64, 66, 69, 70, 80]; therefore, additional approaches to increase the number of eligible patients and/or decrease the risk of relapse after discontinuation, such as combination therapy, are still under development. In the case of relapse, the threshold for restarting treatment remains under investigation; early clinical trials used MR^4.5^ as the cutoff for treatment reintroduction [69], whereas later clinical trials used MR^4^ or even MMR [61, 62, 64, 66, 70]. Data from ENESTfreedom showed that most of the patients who lost MR^4^ also lost MMR after further follow-up; therefore, the loss of MMR or confirmed MR^4^ is a reasonable cutoff for treatment re-initiation.

#### Treatment approach summary

The patient in case study 3 is a female of child-bearing potential who achieved sustained DMR with 1L imatinib, with a special interest in discontinuing treatment; therefore, she is a candidate for TFR. Data from various studies (STIM, A-STIM, EURO-SKI, DESTINY) suggest that there is approximately a 50–60% probability of maintaining TFR for up to 7 years with imatinib (Table 2) [61, 62, 64, 80]. The patient should be aware of the possibility of relapse and the need for continual monitoring during TFR. However, if a relapse occurs, DMR can be successfully achieved after restarting treatment with imatinib. It should be noted that conception would complicate the re-treatment process and should be considered during treatment decision.

#### Second treatment-free remission attempt (TFR2)

Although there are well documented studies outlining TFR1, limited data are available on TFR2. As shown in Table 2, about half of the patients attempting TFR will relapse, mostly within 6 months of treatment discontinuation [61, 62, 64, 66, 69, 70, 80]; however, in most cases, patients can regain DMR after re-treatment [66, 69, 70], making TFR2 an interesting discussion for clinicians.

Although a TFR2 is possible, studies to date have yielded mixed results. In the ReSTIM trial, 36% of patients had a successful TFR2 after discontinuing treatment for a median of 5 months [83]. However, in the TRAD trial, just 22% of patients remained in TFR2 at 6 months [84]. In both trials, patients who relapsed within 3 months during TFR1 were more likely to relapse during TFR2. A notable difference between the two studies is the duration of DMR prior to TFR2; a longer duration of DMR prior to TFR2 may be considered. However, patients should be informed of a lower probability of a successful TFR2 compared with TFR1, and strict monitoring is required. In case of TFR failure in patients treated with 1L imatinib, switching to a 2G TKI before TFR2 could be one of several reasonable strategies for patients with a deep motivation for TFR. Therefore, if the patient in case study 3 were to relapse, a 2G TKI—although not tested prospectively—could be considered when restarting treatment to attempt a deeper, more durable response; clinical trials may be an alternative option.

Clinical trials exploring combination therapy after relapse from TFR1 are currently in progress. An ongoing trial is evaluating the addition of ruxolitinib to available first-/second-generation TKIs after relapse from TFR1 with the aim of increasing the probability of a successful TFR2 (NCT03610971). A similar trial investigating the addition of asciminib to imatinib in patients treated with imatinib who had experienced relapse post-TFR1 is ongoing (NCT04838041).

## COVID-19

The COVID-19 pandemic has greatly affected clinical practice, monitoring, and treatment of cancer in general, including CML. Due to preventive measures, access to the clinic may be limited or adapted for remote care, meaning patients may not be visiting the clinic regularly and may require modified methods to be diagnosed. Thus, there is a risk of delayed recognition of lack of response and/or intolerance, or in the worst case scenario, delaying care until the disease is at a more advanced stage. Moreover, patients attempting TFR may face challenges in attending appointments for regular monitoring, which may delay the timing for treatment re-initiation and increase the risk of recurrence/progression. Despite suggestions from preliminary studies [85], there is no evidence to date that TKI therapy can have a protective effect for patients with CML from SARS-CoV-2 infection or can worsen outcomes for patients who are infected with SARS-CoV-2.

The American Society of Hematology and International CML Foundation have released a series of guidelines based on worldwide experience for patients and clinicians [86, 87]. Patients with ND CML-CP should be monitored and treated as per standard protocol, and patients with CML-CP already undergoing TKI therapy should continue their current regimen. In the event of being infected with SARS-CoV-2, TKI therapy should be continued. Where possible, to minimize the risk of infection with SARS-CoV-2, *BCR*::*ABL1* monitoring of patients should be done remotely via at-home sample collection kits.

To date, guidelines recommend COVID-19 vaccination after discussion with the patient’s healthcare team. Generally, patients with CML may not be immunocompromised, and available data suggest a good immune response to COVID-19 vaccines. Expert recommendations have been published elsewhere [88].

## New/future treatment approaches

New treatments are being developed for heavily pretreated patients and for those who are intolerant or have experienced resistance or disease progression with approved therapies. The US Food and Drug administration recently approved asciminib, a novel, first-in-class STAMP inhibitor, that is effective against the multi-TKI-resistant T315I mutation [89].

The efficacy of asciminib in patients who had ≥ 2 prior TKIs has been shown in the phase 3 ASCEMBL trial [90]; patients treated with asciminib (two doses of 40 mg per day) demonstrated statistically significant improvement in MMR at 24 weeks compared with bosutinib (25.5% vs. 13.2%, 2-side *P* = 0.029), with thrombocytopenia and neutropenia being the most common AEs associated with asciminib. Also, hypertension was observed at a higher rate in patients treated with asciminib compared with bosutinib (11.5% vs. 3.9%), and five patients (3.2%) treated with asciminib experienced AOEs (two fatal) compared with one patient treated with bosutinib (1.3%) [90]. Mutations conferring resistance to asciminib developed rarely during in vivo testing; in addition, based on its distinct mechanism of action targeting the myristoyl pocket, asciminib in combination with TKIs targeting the ATPase domain of *BCR*::*ABL1* has been shown to help suppress the emergence of resistance [91–93] and warrants further investigation. Also, asciminib does not appear to be effective against certain *BCR*::*ABL*^T315I^ and *BCR*::*ABL*^F359I^ mutations as a single agent; therefore, combination therapy may be required for some patients [91]. Comparison between asciminib and ponatinib would be of significant interest.

Although not a new agent, an adapted schedule of administration (response-directed dose reduction) has been used for ponatinib in the OPTIC trial [94], which may decrease the risk of AOEs. This response-adapted approach can be considered for overall treatment with TKIs in the future.

In addition, there are other potential agents for patients who experience treatment failure or intolerance to dasatinib, nilotinib, or bosutinib (Table 3). These include the 2G TKIs radotinib and flumatinib, both of which have shown improved efficacy over imatinib in ND CML-CP in phase 3 clinical trials with tolerable safety profiles [95, 96], and are being assessed as potential 2L options in patients with CML-CP resistant or intolerant to 1L therapy; the third-generation TKIs vodobatinib and olverembatinib [97–100]; and PF-114, a potent TKI that has demonstrated efficacy in a phase 1 trial in patients with CML-CP who have previously been treated with at least two therapies or patients with the T315I mutation who have been treated for ≥ 6 months [101]. The continued emergence of new therapies is welcomed and will change the way clinicians treat CML in the future.

**Table 3:** Summary of future treatment landscape

Key trial information	Key efficacy	Key safety
*2G TKIs*		
*Radotinib*: 2G TKI with activity against native and kinase-domain mutant *BCR*::*ABL*, currently undergoing testing to assess efficacy in CML-CP with failure or intolerance to prior TKI therapy [106]		
*RERISE* (NCT01511289) [107]: phase 3 trial comparing radotinib with imatinib in patients with ND CML-CP in the Republic of Korea, Indonesia, the Philippines, and Thailand	MMR at 12 months (primary endpoint): Radotinib 300 mg BID 52% (*P* = 0.0044 vs. imatinib) Radotinib 400 mg BID 46% (*P* = 0.0342 vs. imatinib) Imatinib 30% CCyR at 12 months (secondary endpoint): Radotinib 300 mg BID 91% (*P* = 0.0120 vs. imatinib) Radotinib 400 mg BID 82% (not significant vs. imatinib) Imatinib 77%	Grade 3–4 neutropenia was the most frequently reported hematologic AE: Radotinib 300 mg 19% Radotinib 400 mg 23% Imatinib 32%
Phase 3 multinational (Republic of Korea, Turkey, Russian Federation, and Ukraine) trial to assess efficacy in CML-CP with failure or intolerance to prior TKI therapy (NCT03459534; currently recruiting)	Data not yet available.	Data not yet available.
*Flumatinib*: imatinib derivative that displays increased efficacy over imatinib in Chinese patients with ND CML-CP with a similar safety profile [96]		
*FESTnd* (NCT02204644) [96]: phase 3 trial: flumatinib vs. imatinib in ND CML-CP	MMR at 6 months (primary endpoint): Flumatinib 34%, imatinib 18% (*P* = 0.0006) EMR at 3 months (secondary endpoint): Flumatinib 82%, imatinib 53% (*P* < 0.0001)	All-grade AEs more frequent in flumatinib arm: Diarrhea (*n* = 79/196, 40%) Alanine transaminase elevation (*n* = 51/196, 26%) All-grade AEs more frequent in imatinib arm: Edema (*n* = 70/198, 35%) Pain in extremities (*n* = 49/198, 25%) Rash (*n* = 28/198, 14%) Neutropenia Thrombocytopenia Anemia Hypophosphatemia
NCT04677439: currently recruiting patients to a phase 4 trial in China: efficacy and safety of flumatinib in patients with Ph + CML-CP post-imatinib failure	Data not yet available.	Data not yet available.
*3G TKIs*		
*Vodobatinib*: novel 3G TKI with limited off-target activity effective against native and mutated *BCR*::*ABL* [108]		
NCT02629692: multinational phase 1/2 trial in ponatinib-treated and naive patients with CML-CP who failed ≥ 3 TKIs (or fewer, if not eligible for other approved 3G TKIs) to determine MTD and RP2D [97]	MTD (primary endpoint): 204 mg Efficacy (secondary endpoint): MMR: 3/16 in ponatinib-treated and 4/15 in ponatinib-naive patients MCyR: 5/16 in ponatinib-treated patients CCyR: 3/15 in ponatinib-naive patients Disease progression: 2/16 in ponatinib-treated and 4/15 in ponatinib-naive patients	TEAEs grade ≥ 3 reported in > 1 ponatinib-treated patient: 2 (13%) each of neutropenia, amylase increase, and thrombocytopenia TEAEs grade ≥ 3 reported in 7 (47%) ponatinib-naive patients: 1 of each: anemia, pneumonia, neutropenia, gout, hypokalemia and thrombocytopenia, dementia, amnesia, and increased liver and pancreatic enzymes
*Olverembatinib*: a novel, broad-spectrum *BCR*::*ABL1* TKI active against T315I mutations [98]		
Phase 1 dose escalation/expansion trial assessing safety, preliminary efficacy, and pharmacokinetic and dynamic properties in Chinese patients with TKI-resistant CML-CP/AP [99]	CHR within 3 cycles (primary endpoint): CML-AP: 58% (*n* = 7/58) MCyR ≥ 3 cycles (primary endpoint): CML-CP: 54% (*n* = 21/58)	≥ 1 grade 3–4 TRAE: 44 (63%) of all patients Dose-limiting toxicities: 2/3 patients in 60 mg cohort
Phase 1 dose escalation/expansion trial to determine maximum tolerated dose and dose-limiting toxicity in Chinese patients with TKI-resistant CML-CP/AP [98]	CHR: CML-CP: 95% (*n* = 52/55) CML-AP: 85% (*n* = 11/13) CCyR: CML-CP: 61% (*n* = 49/81) CML-AP: 36% (*n* = 5/14)	Most common grade ≥ 3 AEs in > 10% patients: Thrombocytopenia (*n* = 50/101, 50%) Leukopenia (*n* = 20/101, 20%) Anemia (*n* = 12/101, 12%)
*4G TKI*		
PF-114: potent 4G TKI selective against native *BCR*::*ABL* and *BCR*::*ABL* harboring the T315I mutation [109]		
NCT02885766: phase 1 trial in patients with CML-CP/AP failing ≥ 2 TKIs or with *BCR*::*ABL1* T315I with ≥ 6 months’ therapy to determine MTD and dose-limiting toxicity. Interim analysis at ≥ 6 months [101]	MTD (primary endpoint): 600 mg Dose-limiting toxicity (primary endpoint): 600 mg manifesting as grade 3 psoriasis-like skin lesions MCyR: 6/11 patients receiving 300 mg dose 4/12 patients with the *BCR*::*ABL1* T315I mutation	Discontinuations due to progression: *n* = 18/51 (35%) Discontinuations due to AEs: *n* = 6/51 (12%) Reversible grade 3 skin toxicity (psoriasis-like skin lesions): 11 patients ≥ 400 mg dose
*STAMP inhibitor*		
*Asciminib*: novel, first-in-class STAMP inhibitor that binds to the myristoyl pocket of *BCR*::*ABL* [91]		
NCT03106779: multicenter phase 3 trial comparing asciminib and bosutinib in patients with CML-CP previously treated with ≥ 2 TKIs [110]	MMR at 24 weeks (primary endpoint) Asciminib 26%; bosutinib 13% (*P* = 0.029)	Grade ≥ 3 TRAEs reported in 51% asciminib- and 61% bosutinib-treated patients 1 patient died due to treatment-related serious AE in the bosutinib arm

*1G* first-generation; *2G* second-generation; *3G* third-generation; *4G*, fourth-generation; *AE* adverse event; *AP* accelerated phase; *BID* twice daily; *CCyR* complete cytogenetic response; *CHR* complete hematologic response; *CML-AP* chronic myeloid leukemia in acute phase; *CML-CP* chronic myeloid leukemia in chronic phase; *EMR* early molecular response; *MCyR* major cytogenetic response; *MMR* major molecular response; *MTD* maximum tolerated dose; *ND* newly diagnosed; *Ph* +  Philadelphia positive; *STAMP* specifically targeting the ABL myristoyl pocket; *TEAE* treatment-emergent adverse event; *TKI* tyrosine kinase inhibitor; and *TRAE* treatment-related adverse event

## Conclusions

With more approved TKIs being available, treatment decisions have become more complex. Treatment choice in the 1L setting is not only influenced by efficacy and safety of the TKIs, but also by patient-specific factors and real-world considerations. Patient choice and circumstances are also increasingly impacting treatment strategies. Regular monitoring to inform treatment options in the event of treatment failure/intolerance to 1L therapy and early switching has been shown to improve responses in patients. Improved efficacy with 2G TKIs has led to increased likelihood to achieve DMR; thus, TFR is quickly becoming a treatment goal for patients. More patients treated with 2G TKIs achieve TFR than patients with imatinib; in most cases, patients who relapsed remained sensitive to TKIs, regaining MMR upon re-treatment. Because patients in TFR can relapse, a better understanding of a second TFR is important to help inform treatment decisions. In addition, management of CML during the COVID-19 pandemic has been challenging, but the release of guidelines and recommendations on treatment continuation and vaccination have helped to guide clinicians and patients. Finally, the recent emergence of new therapies is expanding treatment options for patients with CML, especially those with the T315I mutation.

## Data Availability

Not applicable.

## Abbreviations

1G: First-generation
1L: First-line
2G: Second-generation
2L: Second-line
3G: Third-generation
4G: Fourth-generation
AE: Adverse event
AOE: Arterio-occlusive event
AP: Accelerated phase
BID: Twice daily
CCI: Charlson Comorbidity Index
CCyR: Complete cytogenetic response
CHR: Complete hematologic response
CML: Chronic myeloid leukemia
CML-CP: Chronic myeloid leukemia in chronic phase
CMR: Complete medical response
CV: Cardiovascular
DMR: Deep molecular response
ECOG PS: Eastern Cooperative Oncology Group performance status
ELN: European LeukemiaNet
ELTS: European Treatment and Outcome Study long-term survival
EMR: Early molecular response
MCyR: Major cytogenetic response
MMR: Major molecular response
MR^4^: 4-Log reduction in *BCR*::*ABL1* levels
MR^4.5^: 4.5-Log reduction in *BCR*::*ABL1* levels
mRFS: Molecular relapse-free survival
MTD: Maximum tolerated dose
NCCN: National Comprehensive Cancer Network
ND: Newly diagnosed
Ph+: Philadelphia positive
TEAE: Treatment-emergent adverse event
TFR: Treatment-free remission
TFR1: First treatment-free remission attempt
TFR2: Second treatment-free remission attempt
TKI: Tyrosine kinase inhibitor
TRAE: Treatment-related adverse event

## References

[1] Lin Q, Mao L, Shao L, Zhu L, Han Q, Zhu H (2020). Global, regional, and national burden of chronic myeloid leukemia, 1990–2017: a systematic analysis for the global burden of disease study 2017. Front Oncol..

[2] Kantarjian H, O'Brien S, Jabbour E, Garcia-Manero G, Quintas-Cardama A, Shan J (2012). Improved survival in chronic myeloid leukemia since the introduction of imatinib therapy: a single-institution historical experience. Blood.

[3] ARIAD Pharmaceuticals (2022). Iclusig (ponatinib) [prescribing information].

[4] Novartis Pharmaceuticals (2021). Tasigna (nilotinib) [prescribing information].

[5] Bristol Myers Squibb (2021). Sprycel (dasatinib) [prescribing information].

[6] Pfizer Inc (2021). Bosulif (bosutinib) [prescribing information].

[7] Novartis Pharmaceuticals (2020). Gleevec (imatinib) [prescribing information].

[8] Bower H, Bjorkholm M, Dickman PW, Hoglund M, Lambert PC, Andersson TM (2016). Life expectancy of patients with chronic myeloid leukemia approaches the life expectancy of the general population. J Clin Oncol.

[9] Sasaki K, Strom SS, O'Brien S, Jabbour E, Ravandi F, Konopleva M (2015). Relative survival in patients with chronic-phase chronic myeloid leukaemia in the tyrosine-kinase inhibitor era: analysis of patient data from six prospective clinical trials. Lancet Haematol.

[10] Delord M, Foulon S, Cayuela JM, Rousselot P, Bonastre J (2018). The rising prevalence of chronic myeloid leukemia in France. Leuk Res.

[11] Hochhaus A, Baccarani M, Silver RT, Schiffer C, Apperley JF, Cervantes F (2020). European LeukemiaNet 2020 recommendations for treating chronic myeloid leukemia. Leukemia.

[12] Sato E, Iriyama N, Tokuhira M, Takaku T, Ishikawa M, Nakazato T (2020). The EUTOS long-term survival score predicts disease-specific mortality and molecular responses among patients with chronic myeloid leukemia in a practice-based cohort. Cancer Med.

[13] National Comprehensive Cancer Network. NCCN clinical practice guidelines in oncology: chronic myeloid leukemia (v3.2021). Available from: https://www.nccn.org/professionals/physician_gls/pdf/cml.pdf. Accessed 4 Mar 2021.

[14] Kantarjian H, Shah NP, Hochhaus A, Cortes J, Shah S, Ayala M (2010). Dasatinib versus imatinib in newly diagnosed chronic-phase chronic myeloid leukemia. N Engl J Med.

[15] Gambacorti-Passerini C, Deininger MW, Mauro MJ, Chuah C, Kim D-W, Dyagil I (2017). Bosutinib vs imatinib for newly diagnosed chronic myeloid leukemia (CML) in the BFORE Trial: 18 month follow-up. Blood.

[16] Saglio G, Kim DW, Issaragrisil S, le Coutre P, Etienne G, Lobo C (2010). Nilotinib versus imatinib for newly diagnosed chronic myeloid leukemia. N Engl J Med.

[17] Cortes JE, Saglio G, Kantarjian HM, Baccarani M, Mayer J, Boque C (2016). Final 5-year study results of DASISION: the dasatinib versus imatinib study in treatment-naive chronic myeloid leukemia patients trial. J Clin Oncol.

[18] Hochhaus A, Saglio G, Hughes TP, Larson RA, Kim DW, Issaragrisil S (2016). Long-term benefits and risks of frontline nilotinib vs imatinib for chronic myeloid leukemia in chronic phase: 5-year update of the randomized ENESTnd trial. Leukemia.

[19] Brümmendorf TH, Cortes JE, Milojkovic D, Gambacorti-Passerini C, Clark RE, le Coutre PD, et al. Bosutinib (BOS) versus imatinib for newly diagnosed chronic phase (CP) chronic myeloid leukemia (CML): final 5-year results from the BFORE trial. American Society of Hematology 2020. December 5, 2020:Oral presentation 46.

[20] Kantarjian HM, Hughes TP, Larson RA, Kim DW, Issaragrisil S, le Coutre P (2021). Long-term outcomes with frontline nilotinib versus imatinib in newly diagnosed chronic myeloid leukemia in chronic phase: ENESTnd 10-year analysis. Leukemia.

[21] Pemmaraju N, Kantarjian H, Shan J, Jabbour E, Quintas-Cardama A, Verstovsek S (2012). Analysis of outcomes in adolescents and young adults with chronic myelogenous leukemia treated with upfront tyrosine kinase inhibitor therapy. Haematologica.

[22] Montoriol-Sabaté C, Martínez-Laperche C, Jiménez-Gámiz P, Collado R, Minguela-Puras A, Piñán-Francés M (2013). Chronic myeloid leukemia (CML) patients with atypical e1a2 P190 BCR-ABL translocation show a poor response to therapy with tyrosine kinase inhibitors (TKI). Blood.

[23] Saußele S, Krauß M-P, Hehlmann R, Lauseker M, Proetel U, Kalmanti L (2015). Impact of comorbidities on overall survival in patients with chronic myeloid leukemia: results of the randomized CML Study IV. Blood.

[24] Breccia M, Mauro MJ, Jabbour E, Saglio G, Jimenez-Velasco A, le Coutre PD, et al. Impact of comorbidities on response outcomes in patients with chronic myeloid leukemia in chronic phase treated with first-line dasatinib versus imatinib: exploratory post hoc analysis of DASISION. American Society of Hematology 2020. December 7, 2020:Oral presentation 3074.

[25] Cortes JE, Gambacorti-Passerini C, Deininger MW, Mauro MJ, Chuah C, Kim DW (2018). Bosutinib versus imatinib for newly diagnosed chronic myeloid leukemia: results from the randomized BFORE trial. J Clin Oncol.

[26] Quintas-Cardama A, Kantarjian H, O'Brien S, Borthakur G, Bruzzi J, Munden R (2007). Pleural effusion in patients with chronic myelogenous leukemia treated with dasatinib after imatinib failure. J Clin Oncol.

[27] de Lavallade H, Punnialingam S, Milojkovic D, Bua M, Khorashad JS, Gabriel IH (2008). Pleural effusions in patients with chronic myeloid leukaemia treated with dasatinib may have an immune-mediated pathogenesis. Br J Haematol.

[28] Bhupathiraju SN, Hu FB (2016). Epidemiology of obesity and diabetes and their cardiovascular complications. Circ Res.

[29] Breccia M, Loglisci G, Salaroli A, Serrao A, Mancini M, Diverio D (2013). Delayed cytogenetic and major molecular responses associated to increased BMI at baseline in chronic myeloid leukemia patients treated with imatinib. Cancer Lett.

[30] Breccia M, Cortes JE, Shah NP, Saglio G, Jiménez-Velasco A, Le Coutre P (2019). Association of high body mass index with response outcomes in patients with CML-CP treated with dasatinib versus imatinib in the first line: exploratory post hoc analysis of the phase 3 DASISION trial. Blood.

[31] Brümmendorf TH, Cortes JE, Busque L, Gambacorti-Passerini C, Stenke L, Viqueira A (2021). The effect of body mass index on efficacy and safety of bosutinib or imatinib in patients with newly diagnosed chronic myeloid leukemia. J Clin Oncol.

[32] Delphine R, Gautier J-f, Breccia M, Saglio G, Hughes TP, Kantarjian HM (2012). Incidence of hyperglycemia by 3 years in patients (pts) with newly diagnosed chronic myeloid leukemia in chronic phase (CML-CP) treated with nilotinib (NIL) or imatinib (IM) in ENESTnd. Blood.

[33] Cortes JE, Gambacorti-Passerini C, Kim DW, Kantarjian HM, Lipton JH, Lahoti A (2017). Effects of bosutinib treatment on renal function in patients with philadelphia chromosome-positive leukemias. Clin Lymphoma Myeloma Leuk.

[34] Ganesan P, Kumar L (2017). Chronic myeloid leukemia in India. J Glob Oncol.

[35] Gupta N, Mahapatra M, Seth T, Tyagi S, Sazawal S, Saxena R (2021). Social and financial barriers to optimum TKI treatment in patients with chronic myeloid leukemia–a knowledge-attitudes-practices study from India. Mediterr J Hematol Infect Dis.

[36] Marin D, Bazeos A, Mahon FX, Eliasson L, Milojkovic D, Bua M (2010). Adherence is the critical factor for achieving molecular responses in patients with chronic myeloid leukemia who achieve complete cytogenetic responses on imatinib. J Clin Oncol.

[37] Ganesan P, Ganesan TS, Radhakrishnan V, Sagar TG, Kannan K, Dhanushkodi M (2019). Chronic myeloid leukemia: long-term outcome data in the imatinib era. Indian J Hematol Blood Transfus.

[38] Phuar HL, Begley CE, Chan W, Krause TM (2020). Tyrosine kinase inhibitors and the relationship with adherence, costs, and health care utilization in commercially insured patients with newly diagnosed chronic myeloid leukemia: a retrospective claims-based study. Am J Clin Oncol.

[39] Wu EQ, Johnson S, Beaulieu N, Arana M, Bollu V, Guo A (2010). Healthcare resource utilization and costs associated with non-adherence to imatinib treatment in chronic myeloid leukemia patients. Curr Med Res Opin.

[40] Ector G, Govers TM, Westerweel PE, Grutters JPC, Blijlevens NMA (2019). The potential health gain and cost savings of improving adherence in chronic myeloid leukemia. Leuk Lymphoma.

[41] Darkow T, Henk HJ, Thomas SK, Feng W, Baladi JF, Goldberg GA (2007). Treatment interruptions and non-adherence with imatinib and associated healthcare costs: a retrospective analysis among managed care patients with chronic myelogenous leukaemia. Pharmacoeconomics.

[42] Latremouille-Viau D, Guerin A, Gagnon-Sanschagrin P, Dea K, Cohen BG, Joseph GJ (2017). Health care resource utilization and costs in patients with chronic myeloid leukemia with better adherence to tyrosine kinase inhibitors and increased molecular monitoring frequency. J Manag Care Spec Pharm.

[43] Noens L, Hensen M, Kucmin-Bemelmans I, Lofgren C, Gilloteau I, Vrijens B (2014). Measurement of adherence to BCR-ABL inhibitor therapy in chronic myeloid leukemia: current situation and future challenges. Haematologica.

[44] Boons CCLM, Timmers L, Janssen JJWM, Westerweel PE, Blijlevens NMA, Smit WM (2020). Response and Adherence to Nilotinib in Daily practice (RAND study): an in-depth observational study of chronic myeloid leukemia patients treated with nilotinib. Eur J Clin Pharmacol.

[45] Geissler J, Sharf G, Bombaci F, Daban M, De Jong J, Gavin T (2017). Factors influencing adherence in CML and ways to improvement: results of a patient-driven survey of 2546 patients in 63 countries. J Cancer Res Clin Oncol.

[46] Dusetzina SB, Winn AN, Abel GA, Huskamp HA, Keating NL (2014). Cost sharing and adherence to tyrosine kinase inhibitors for patients with chronic myeloid leukemia. J Clin Oncol.

[47] Quintás-Cardama A, Jabbour EJ (2013). Considerations for early switch to nilotinib or dasatinib in patients with chronic myeloid leukemia with inadequate response to first-line imatinib. Leuk Res.

[48] Quintas-Cardama A, Kantarjian H, Jones D, Shan J, Borthakur G, Thomas D (2009). Delayed achievement of cytogenetic and molecular response is associated with increased risk of progression among patients with chronic myeloid leukemia in early chronic phase receiving high-dose or standard-dose imatinib therapy. Blood.

[49] Cortes JE, Jiang Q, Wang J, Weng J, Zhu H, Liu X (2020). Dasatinib vs. imatinib in patients with chronic myeloid leukemia in chronic phase (CML-CP) who have not achieved an optimal response to 3 months of imatinib therapy: the DASCERN randomized study. Leukemia.

[50] Baccarani M, Deininger MW, Rosti G, Hochhaus A, Soverini S, Apperley JF (2013). European LeukemiaNet recommendations for the management of chronic myeloid leukemia: 2013. Blood.

[51] Cortes JE, De Souza CA, Ayala M, Lopez JL, Bullorsky E, Shah S (2016). Switching to nilotinib versus imatinib dose escalation in patients with chronic myeloid leukaemia in chronic phase with suboptimal response to imatinib (LASOR): a randomised, open-label trial. Lancet Haematol.

[52] Hughes TP, Saglio G, Kantarjian HM, Guilhot F, Niederwieser D, Rosti G (2014). Early molecular response predicts outcomes in patients with chronic myeloid leukemia in chronic phase treated with frontline nilotinib or imatinib. Blood.

[53] Milojkovic D, Cross NCP, Ali S, Byrne J, Campbell G, Dignan FL (2021). Real-world tyrosine kinase inhibitor treatment pathways, monitoring patterns and responses in patients with chronic myeloid leukaemia in the United Kingdom: the UK TARGET CML study. Br J Haematol.

[54] Hehlmann R, Cortes JE, Zyczynski T, Gambacorti-Passerini C, Goldberg SL, Mauro MJ (2019). Tyrosine kinase inhibitor interruptions, discontinuations and switching in patients with chronic-phase chronic myeloid leukemia in routine clinical practice: SIMPLICITY. Am J Hematol.

[55] Goldberg S, Michallet M, Hehlmann R, Zyczynski T, Foreman A, Calimlim B (2016). Tyrosine kinase inhibitor (TKI) switching patterns during the first 12 months in SIMPLICITY, an observational study of chronic-phase chronic myeloid leukemia (CP-CML) tatients (Pts) in routine clinical practice. Blood.

[56] Breccia M, Olimpieri PP, Olimpieri O, Pane F, Iurlo A, Foggi P (2020). How many chronic myeloid leukemia patients who started a frontline second-generation tyrosine kinase inhibitor have to switch to a second-line treatment? A retrospective analysis from the monitoring registries of the Italian medicines agency (AIFA). Cancer Med.

[57] Breccia M, Luciano L, Annunziata M, Attolico I, Malato A, Abruzzese E (2021). Multicenter, prospective and retrospective observational cohort study of ponatinib in patients with CML in Italy: primary analysis of the OITI trial. Blood.

[58] Cortes JE, Kim DW, Pinilla-Ibarz J, le Coutre PD, Paquette R, Chuah C (2018). Ponatinib efficacy and safety in Philadelphia chromosome-positive leukemia: final 5-year results of the phase 2 PACE trial. Blood.

[59] Cortes JE, Apperley J, Lomaia E, Moiraghi B, Sutton MU, Pavlovsky C (2021). OPTIC primary analysis: A dose-optimization study of 3 starting doses of ponatinib (PON). J Clin Oncol.

[60] Baccarani M, Pileri S, Steegmann JL, Muller M, Soverini S, Dreyling M (2012). Chronic myeloid leukemia: ESMO Clinical Practice Guidelines for diagnosis, treatment and follow-up. Ann Oncol.

[61] Saussele S, Richter J, Guilhot J, Gruber FX, Hjorth-Hansen H, Almeida A (2018). Discontinuation of tyrosine kinase inhibitor therapy in chronic myeloid leukaemia (EURO-SKI): a prespecified interim analysis of a prospective, multicentre, non-randomised, trial. Lancet Oncol.

[62] Etienne G, Guilhot J, Rea D, Rigal-Huguet F, Nicolini F, Charbonnier A (2017). Long-term follow-up of the French Stop Imatinib (STIM1) study in patients with chronic myeloid leukemia. J Clin Oncol.

[63] Ross DM, Branford S, Seymour JF, Schwarer AP, Arthur C, Yeung DT (2013). Safety and efficacy of imatinib cessation for CML patients with stable undetectable minimal residual disease: results from the TWISTER study. Blood.

[64] Rousselot P, Loiseau C, Delord M, Cayuela JM, Spentchian M (2020). Late molecular recurrences in patients with chronic myeloid leukemia experiencing treatment-free remission. Blood Adv.

[65] Chamoun K, Kantarjian H, Atallah R, Gonzalez GN, Issa GC, Rios MB (2019). Tyrosine kinase inhibitor discontinuation in patients with chronic myeloid leukemia: a single-institution experience. J Hematol Oncol.

[66] Shah NP, García-Gutiérrez V, Jiménez-Velasco A, Larson S, Saussele S, Rea D (2020). Dasatinib discontinuation in patients with chronic-phase chronic myeloid leukemia and stable deep molecular response: the DASFREE study. Leuk Lymphoma.

[67] Kumagai T, Nakaseko C, Nishiwaki K, Yoshida C, Ohashi K, Takezako N (2018). Dasatinib cessation after deep molecular response exceeding 2 years and natural killer cell transition during dasatinib consolidation. Cancer Sci.

[68] Okada M, Imagawa J, Tanaka H, Nakamae H, Hino M, Murai K (2018). Final 3-year results of the dasatinib discontinuation trial in patients with chronic myeloid leukemia who received dasatinib as a second-line treatment. Clin Lymphoma Myeloma Leuk.

[69] Mahon FX, Boquimpani C, Kim DW, Benyamini N, Clementino NCD, Shuvaev V (2018). Treatment-free remission after second-line nilotinib treatment in patients with chronic myeloid leukemia in chronic phase: results from a single-group, phase 2, open-label study. Ann Intern Med.

[70] Ross DM, Masszi T, Gómez Casares MT, Hellmann A, Stentoft J, Conneally E (2018). Durable treatment-free remission in patients with chronic myeloid leukemia in chronic phase following frontline nilotinib: 96-week update of the ENESTfreedom study. J Cancer Res Clin Oncol.

[71] Rea D, Nicolini FE, Tulliez M, Guilhot F, Guilhot J, Guerci-Bresler A (2017). Discontinuation of dasatinib or nilotinib in chronic myeloid leukemia: interim analysis of the STOP 2G-TKI study. Blood.

[72] Alfayez M, Richard-Carpentier G, Jabbour E, Vishnu P, Naqvi K, Sasaki K (2019). Sudden blastic transformation in treatment-free remission chronic myeloid leukaemia. Br J Haematol.

[73] Breccia M, Abruzzese E, Annunziata M, Luciano L, Sica S (2021). Clinical and psychological factors to consider in achieving treatment-free remission in patients with chronic myeloid leukemia. Front Oncol.

[74] Schoenbeck KL, Atallah E, Lin L, Weinfurt KP, Cortes J, Deininger MWN (2021). Patient-reported functional outcomes in patients with chronic myeloid leukemia after stopping tyrosine kinase inhibitors. J Natl Cancer Inst.

[75] Mori S, Vagge E, le Coutre P, Abruzzese E, Martino B, Pungolino E (2015). Age and dPCR can predict relapse in CML patients who discontinued imatinib: the ISAV study. Am J Hematol.

[76] Shah NP, Garcia-Gutierrez V, Jimenez-Velasco A, Larson S, Saussele S, Rea D (2020). Dasatinib discontinuation in patients with chronic-phase chronic myeloid leukemia and stable deep molecular response: the DASFREE study. Leuk Lymphoma.

[77] Radich JP, Hochhaus A, Masszi T, Hellmann A, Stentoft J, Casares MTG (2021). Treatment-free remission following frontline nilotinib in patients with chronic phase chronic myeloid leukemia: 5-year update of the ENESTfreedom trial. Leukemia.

[78] Wang WJ, Zheng CF, Liu Z, Tan YH, Chen XH, Zhao BL (2018). Droplet digital PCR for BCR/ABL(P210) detection of chronic myeloid leukemia: a high sensitive method of the minimal residual disease and disease progression. Eur J Haematol.

[79] Colafigli G, Scalzulli E, Porrazzo M, Diverio D, Loglisci MG, Latagliata R (2019). Digital droplet PCR at the time of TKI discontinuation in chronic-phase chronic myeloid leukemia patients is predictive of treatment-free remission outcome. Hematol Oncol.

[80] Clark RE, Polydoros F, Apperley JF, Milojkovic D, Rothwell K, Pocock C (2019). De-escalation of tyrosine kinase inhibitor therapy before complete treatment discontinuation in patients with chronic myeloid leukaemia (DESTINY): a non-randomised, phase 2 trial. Lancet Haematol.

[81] Vigón L, Luna A, Galán M, Rodríguez-Mora S, Fuertes D, Mateos E (2021). Identification of immunological parameters as predictive biomarkers of relapse in patients with chronic myeloid leukemia on treatment-free remission. J Clin Med.

[82] Garcia-Gutiérrez V, Vigon L, Checa L, Luna A, Piris-Villaespesa M, Rodriguez-Mora S (2019). Identification of immunological parameters related to relapse in patients with chronic myeloid leukemia on treatment-free remission. Blood.

[83] Legros L, Nicolini FE, Etienne G, Rousselot P, Rea D, Giraudier S (2017). Second tyrosine kinase inhibitor discontinuation attempt in patients with chronic myeloid leukemia. Cancer.

[84] Kim DDH, Busque L, Forrest DL, Savoie L, Bence-Bruckler I, Couban S (2018). Second attempt of TKI discontinuation with dasatinib for treatment-free remission after failing first attempt with imatinib: treatment-free remission accomplished by dasatinib (TRAD) trial. Blood.

[85] Galimberti S, Petrini M, Baratè C, Ricci F, Balducci S, Grassi S (2020). Tyrosine kinase inhibitors play an antiviral action in patients affected by chronic myeloid leukemia: a possible model supporting their use in the fight against SARS-CoV-2. Front Oncol.

[86] American Society of Hematology. COVID-19 and CML: Frequently Asked Questions.2021. Available from: https://www.hematology.org/covid-19/covid-19-and-cml. Accessed 27 Sept 2021.

[87] International Chronic Myeloid Leukemia Foundation. iCMLf COVID-19 Hub.2021. Available from: https://www.cml-foundation.org/covid-19-hub.html. Accessed 27 Sept 2021.

[88] Karki NR, Le T, Cortes J (2021). The care of the leukemic patients in times of SARS-CoV-2. Curr Oncol Rep.

[89] Hughes TP, Mauro MJ, Cortes JE, Minami H, Rea D, DeAngelo DJ (2019). Asciminib in chronic myeloid leukemia after ABL kinase inhibitor failure. N Engl J Med.

[90] Réa D, Mauro MJ, Boquimpani C, Minami Y, Lomaia E, Voloshin S (2021). A phase 3, open-label, randomized study of asciminib, a STAMP inhibitor, vs bosutinib in CML after 2 or more prior TKIs. Blood.

[91] Eide CA, Zabriskie MS, Savage Stevens SL, Antelope O, Vellore NA, Than H (2019). Combining the allosteric inhibitor asciminib with ponatinib suppresses emergence of and restores efficacy against highly resistant BCR-ABL1 mutants. Cancer Cell.

[92] Mauro M, Kim D-W, Cortes J, Réa D, Hughes T, Minami H, et al. Combination of asciminib plus nilotinib (nil) or dasatinib (das) in patients (pts) with chronic myeloid leukemia (cml): results from a phase 1 study. European Hematology Association 2019. June 15, 2019:Oral presentation S884.

[93] Wylie AA, Schoepfer J, Jahnke W, Cowan-Jacob SW, Loo A, Furet P (2017). The allosteric inhibitor ABL001 enables dual targeting of BCR-ABL1. Nature.

[94] Cortes J, Apperley J, Lomaia E, Moiraghi B, Undurraga Sutton M, Pavlovsky C (2021). Ponatinib dose-ranging study in chronic-phase chronic myeloid leukemia: a randomized, open-label phase 2 clinical trial. Blood.

[95] Do YR, Kwak JY, Kim JA, Kim HJ, Chung JS, Shin HJ (2020). Long-term data from a phase 3 study of radotinib versus imatinib in patients with newly diagnosed, chronic myeloid leukaemia in the chronic phase (RERISE). Br J Haematol.

[96] Zhang L, Meng L, Liu B, Zhang Y, Zhu H, Cui J (2021). Flumatinib versus imatinib for newly diagnosed chronic phase chronic myeloid leukemia: a phase III, randomized, open-label, multi-center FESTnd study. Clin Cancer Res.

[97] Cortes JE, Saikia T, Kim D-W, Alvarado Y, Nicolini FE, Khattry N (2020). Phase 1 trial of vodobatinib, a novel oral BCR-ABL1 tyrosine kinase inhibitor (TKI): activity in CML chronic phase patients failing TKI therapies including ponatinib. Blood.

[98] Jiang Q, Huang X, Chen Z, Niu Q, Men L, Wang H (2019). An updated safety and efficacy results of phase 1 study of HQP1351, a novel 3rd generation of BCR-ABL tyrosine kinase inhibitor (TKI), in patients with TKI resistant chronic myeloid leukemia. Blood.

[99] Jiang Q, Huang XJ, Chen Z, Men L, Liu W, Sun X (2018). Safety and efficacy of HQP1351, a 3rd generation oral BCR-ABL inhibitor in patients with tyrosine kinase inhibitor–resistant chronic myelogenous leukemia: preliminary results of phase I study. Blood.

[100] CISION PR Newswire. Ascentage Pharma's Core Drug Candidate HQP1351 Granted Fast Track Designation by the US FDA, Marking Another Milestone in Its Development.2020. Available from: https://www.prnewswire.com/news-releases/ascentage-pharmas-core-drug-candidate-hqp1351-granted-fast-track-designation-by-the-us-fda-marking-another-milestone-in-its-development-301054701.html. Accessed 2 Feb 2022.

[101] Turkina AG, Vinogradova O, Lomaia E, Shatokhina E, Shukhov OA, Chelysheva EY (2019). PF-114: A 4th generation tyrosine kinase-inhibitor for chronic phase chronic myeloid leukaemia including BCRABL1T315I. Blood.

[102] Cortes JE, Mauro MJ, Deininger MWN, Chuah C, Kim D-W, Kota V (2018). Bosutinib vs imatinib for newly diagnosed chronic myeloid leukemia in the BFORE trial: 24-month follow-up. J Clin Oncol.

[103] Hochhaus A, Gambacorti-Passerini C, Deininger MW, Mauro MJ, Chuah C, Kim D-W (2017). Bosutinib vs imatinib for newly diagnosed chronic myeloid leukemia in the BFORE trial: results by 3-month BCR-ABL1 transcript level. Blood.

[104] Matsumura I, Ohtaake S, Atsuta Y, Kurata M, Minami Y, Takahashi N, Nakaseko C, Iriyama N (2020). Nilotinib vs dasatinib in achieving MR^4.5^ for newly diagnosed chronic myeloid leukemia: results of the prospective randomized phase 3 study. JALSG CML212. Blood.

[105] Ritchie EK, Catchatourian R, Klisovic RB, Pinilla-Ibarz J, Deininger MW, Erba HP (2017). Results from Enestgoal: a phase 2 study of treatment-free remission (TFR) in patients (pts) with chronic myeloid leukemia in chronic phase (CML-CP) who switched from imatinib to nilotinib. Blood.

[106] Zabriskie MS, Vellore NA, Gantz KC, Deininger MW, O'Hare T (2015). Radotinib is an effective inhibitor of native and kinase domain-mutant BCR-ABL1. Leukemia.

[107] Kwak JY, Kim SH, Oh SJ, Zang DY, Kim H, Kim JA (2017). Phase III clinical trial (RERISE study) results of efficacy and safety of radotinib compared with imatinib in newly diagnosed chronic phase chronic myeloid leukemia. Clin Cancer Res.

[108] Antelope O, Vellore NA, Pomicter AD, Patel AB, Van Scoyk A, Clair PM (2019). BCR-ABL1 tyrosine kinase inhibitor K0706 exhibits preclinical activity in Philadelphia chromosome-positive leukemia. Exp Hematol.

[109] Mian AA, Rafiei A, Haberbosch I, Zeifman A, Titov I, Stroylov V (2015). PF-114, a potent and selective inhibitor of native and mutated BCR/ABL is active against Philadelphia chromosome-positive (Ph+) leukemias harboring the T315I mutation. Leukemia.

[110] Hochhaus A, Boquimpani C, Rea D, Minami Y, Lomaia E, Voloshin S (2020). Efficacy and safety results from ASCEMBL, a multicenter, open-label, phase 3 study of asciminib, a first-in-class STAMP inhibitor, vs bosutinib (BOS) in patients (pts) with chronic myeloid leukemia in chronic phase (CML-CP) previously treated with ≥2 tyrosine kinase inhibitors (TKIs). Blood.

